# Is governance, gross domestic product, inequality, population size or country surface area associated with coverage and equity of health interventions? Ecological analyses of cross-sectional surveys from 80 countries

**DOI:** 10.1136/bmjgh-2017-000437

**Published:** 2017-10-31

**Authors:** Fernando C Wehrmeister, Inácio Crochemore M da Silva, Aluisio J D Barros, Cesar G Victora

**Affiliations:** International Center for Equity in Health, Federal University of Pelotas, Pelotas, Brazil

**Keywords:** maternal health, child health, epidemiology, public health

## Abstract

**Objective:**

To assess associations between national characteristics, including governance indicators, with a proxy for universal health coverage in reproductive, maternal, newborn and child health (RMNCH).

**Design:**

Ecological analysis based on data from national standardised cross-sectional surveys.

**Setting:**

Low-income and middle-income countries with a Demographic and Health Survey or a Multiple Indicator Cluster Survey since 2005.

**Participants:**

1 246 710 mothers and 2 129 212 children from 80 national surveys.

**Exposures of interest:**

Gross domestic product (GDP), country surface area, population, Gini index and six governance indicators (control of corruption, political stability and absence of violence, government effectiveness, regulatory quality, rule of law, and voice and accountability).

**Main outcomes:**

Levels and inequality in the composite coverage index (CCI), a weighted average of eight RMNCH interventions. Relative and absolute inequalities were measured through the concentration index (CIX) and slope index of inequality (SII) for CCI, respectively.

**Results:**

The average values of CCI (70.5% (SD=13.3)), CIX (5.3 (SD=5.1)) and mean slope index (19.8 (SD=14.7)) were calculated. In the unadjusted analysis, all governance variables and GDP were positively associated with the CCI and negatively with inequalities. Country surface showed inverse associations with both inequality indices. After adjustment, among the governance indicators, only political stability and absence of violence was directly related to CCI (β=6.3; 95% CI 3.6 to 9.1; p<0.001) and inversely associated with relative (CIX; β=−1.4; 95% CI −2.4 to −0.4; p=0.007) and absolute (SII; β=−5.3; 95% CI –8.9 to −1.7; p=0.005) inequalities. The strongest associations with governance indicators were found in the poorest wealth quintile. Similar patterns were observed for GDP. Country surface area was inversely related to inequalities on CCI.

**Conclusions:**

Levels and equity in RMNCH interventions are positively associated with political stability and absence of violence, and with GDP, and inversely associated with country surface area.

**Key questions**

**What is already known about this topic?**

- Gross domestic product (GDP) and income have been consistently associated with health outcomes.
- Two studies on governance and reproductive, maternal, newborn and child health (RMNCH) coverage show conflicting results, and only one study in 34 countries assessed the association between governance and coverage inequalities in immunisation.

**What are the new findings?**

- Our findings highlight the negative impact of poor governance, especially of political instability and violence, as determinants of healthcare inequalities for women and children.
- The effects of good governance appear to be strongest among the poorest women and children in each country.
- Countries with high GDP per capita and small surface areas are more likely to show high and equitable coverage.

**Recommendations for policy**

- Country governments, donors and global instutitions should strenght their partnership to achieve a better governance at global level.
- Local governments need to include governance in their agenda to benefit those who have been left behind.

## Introduction

The Millennium Development Goals (MDGs) gave strong emphasis to reducing maternal and child mortality.^1^ Although most low-income and middle-income countries (LMICs) failed to reach the MDG targets, there was important progress in terms of delivering reproductive, maternal, newborn and child health (RMNCH) interventions up to 2015.^2 3^ Yet large coverage gaps persist, both between and within countries. In addition to the monitoring of such gaps, it is important to understand their drivers, that is, which national-level factors affect the levels and inequalities in intervention coverage.

The Countdown to 2015 was a monitoring and accountability initiative that assessed national and subnational progress towards the MDGs. It was recently renamed as Countdown to 2030, within the framework of the Sustainable Development Goals, launched in 2015 with a deadline in 2030.^4^ Countdown pioneered the composite coverage index (CCI) as a summary indicator for RMNCH coverage, based on eight separate interventions along the continuum of care for which coverage data are available from most LMICs.^5^ The CCI is a robust summary indicator for RMNCH intervention coverage, being able to reveal inequality patterns more precisely than standalone coverage indicators, and to predict levels of mortality and undernutrition.^6^

Several studies investigated likely contextual drivers of maternal and child health and well-being, including economic variables such as income or gross domestic product (GDP).^7–9^ However, other contextual indicators such as good governance may add further light on what drives health intervention coverage and health status. Rajkumar and Swaroop,^10^ for example, showed that the simple increase in health spending is not sufficient for improvements in health outcomes in the presence of poor governance.

Good governance has been linked to lower under-5 mortality rates,^11 12^ lower prevalence of HIV/AIDS^13^ and also to better child nutrition.^14^ We located two previous analyses on the role of governance in explaining the coverage of selected RMNCH outcomes,^15 16^ with conflicting results, as well as an analysis of the association between governance and inequalities in vaccine coverage.^16^ A better understanding of the role of governance in determining RMNCH intervention coverage is relevant for governments themselves, and for global players such as funders, and bilateral and international organisations.

We investigate the associations between six governance indicators and the levels and wealth-related inequalities in CCI coverage in 80 LMICs, both before and after adjustment for national-level variables including GDP, the Gini index for income concentration, country surface area and population. We also assess whether governance is more closely associated with coverage among the rich than among the poor.

## Methods

We used data from Demographic and Health Survey (DHS: http://dhsprogram.com/) and Multiple Indicator Cluster Survey (MICS: http://mics.unicef.org/), which are national representative surveys carried out in LMICs. The International Center for Equity in Health, based in Pelotas, Brazil, disaggregated analyses from these surveys.^17 18^ In the present analyses we used data from the most recent publicly available data set from each country from 2005 and onwards.

The outcome variable is the CCI, a weighted average of the coverage of eight interventions along the four stages of the RMNCH continuum of care: reproductive care (demand for family planning satisfied or DFPS), maternal care (at least one antenatal care visit with skilled provider or ANC1, and skilled birth attendance or SBA), immunisation (Bacillus Calmette-Guérin (BCG), three doses of diphteria-tetanus-pertussis (DPT3) and measles (MSL) vaccines) and management of child illness (oral rehydration therapy for diarrhoea or ORT, and care-seeking for symptoms of pneumonia or CPNM). According to the following formula, each stage receives the same weight, and within each stage the indicators are equally weighted (except for DPT3 vaccines that receive a weight of 2 because it requires more than one dose).

$$CCI=\;\frac{1}{4}\;\left(DFPS+\;\frac{ANC1+SBA}{2}+\frac{2DPT3+MSL+BCG}{4}+\;\frac{ORT+CPNM}{2}\right)$$

Trends in CCI were calculated according to wealth and place of residence. A wealth index based on household assets and building characteristics is derived for all households in each survey through a principal component analysis (PCA), according to that proposed by Rutstein and Johnson.^19^ Quintiles derived from the first component of the PCA were used, with Q1 representing the 20% poorest while the Q5 representing the wealthiest 20% of all families. Families were also classified living in urban or rural areas according to country-specific definitions. Our analyses were restricted to surveys with information on the eight coverage indicators that are included in the CCI, plus information on asset indices and residence.

Two summary indices of inequality were calculated, the concentration index (CIX) and the slope index of inequality (SII). The CIX uses an analogous approach compared with the Gini index by ranking individuals according to socioeconomic position on the x-axis and plotting, in the present analyses, cumulative intervention coverage on the y-axis. The CIX is a relative measure of inequality and it might be expressed on a scale from −100 to +100, with 0 representing equal distribution of the attribute across the wealth scale. Positive CIX values represent a pro-rich distribution, usually observed for health coverage indicators, including the CCI.^17^ The SII is a measure of absolute inequality obtained through logistic regression of the health outcome on the midpoints of the ranks obtained by ordering the sample by the explanatory variable when using grouped data.^17^ The SII indicates the difference in percentage points (ranging from −100 to +100) between the fitted values of the health indicator for the top and the bottom of the wealth distribution. As for the CIX, positive values indicate higher coverage among the rich.

The explanatory variables used in the analyses included governance indicators and other national characteristics. All governance indicators were obtained from the World Bank database, being derived from several data sources and summarised in six dimensions: voice and accountability, political stability and absence of violence, government effectiveness, regulatory quality, rule of law, and control of corruption. The following is the definition of governance underlying these indicators, according to the World Bank:

‘the traditions and institutions by which authority in a country is exercised. This includes (a) the process by which governments are selected, monitored and replaced; (b) the capacity of the government to effectively formulate and implement sound policies; and (c) the respect of citizens and the state for the institutions that govern economic and social interactions among them’.

All indicators are expressed in z-scores ranging from −2.5 to +2.5, with higher values indicating good performance in each indicator.^20^ The definition of each governance indicator is shown in online supplementary file 1. More details about the development of the governance indicators are available on the World Bank website.^21^

10.1136/bmjgh-2017-000437.supp1 Supplementary file 1

GDP per capita, Gini coefficient, surface area and country population were also obtained from the World Bank database.^22^ The GDP per capita is expressed in US$ and converted to international dollars using the power purchasing parity rates. Due to the skewed distribution of this variable, we used a log scale transformation for all analyses. The Gini coefficient varies from 0 to 100, with 100 representing complete inequality. When the value of the coefficient was not available for the year when the survey was conducted, we used linear interpolation between the closest years available for the country; if the most recent value was before the survey year, we used this latest value. Surface area is the total area of a given country, expressed in square kilometres, excluding areas under inland water bodies, national claims to continental shelf and exclusive economic zones. Country population considers midyear estimates of all residents regardless of the legal status or citizenship.

Our analyses followed an ecological design with countries as the units. Descriptive statistics were performed using mean and SD. We also calculated Pearson correlation coefficients among all variables. Unadjusted and adjusted meta-regression models were used to account for within-country variability in the coverage estimates, and to obtain regression coefficients adjusted for possible confounders. Adjusted analyses included backward elimination of variables in two steps, first for the six governance indicators and second for other country characteristics. The final adjusted model included all variables that remained with p<0.05 in the previous models.

The initial analyses used the national values for the CCI, and were followed by analyses for wealth quintiles and according to area of residence. All analyses were performed using Stata V.13.1 (StataCorp. 2013. Stata Statistical Software: Release 13. College Station, TX: StataCorp LP).

### Patient involvement

This study is based on data publicly available from national surveys (DHS and MICS). There is no involvement of patients in any phase of the study.

## Results

Data were available for 80 countries. Table 1 shows the descriptive statistics for all variables included in the analyses. The CCI ranged from 33.5 in Chad to 89.8 in Costa Rica. The mean CIX was 5.3 (SD 5.1), ranging from −1.3 (Maldives) to 27.8 (Nigeria). Absolute inequality, expressed as the SII, had a mean value of 19.7 (SD 14.7), with the same countries presenting the lowest and highest (−6.3 and 69.8) values as for CIX. The mean of governance indicators was similar (around −0.6 z-scores), with political stability and absence of violence presenting the highest variability, ranging from −2.7 in Pakistan to 0.9 in Namibia. The mean Gini index for income was 40.5 (SD=7.5), while the GDP per capita, in log scale, ranged from 6.5 (US$639.00) to 9.9 (US$20 521.00). Countries vary widely in area, the smallest being Maldives (300 km^2^) and the largest, India (nearly 3 million km^2^). Similarly, there were huge variations in population size: Sao Tome and Principe was the smallest country (around 164 000), while India was the largest (>1 billion). Detailed country geodemographic information is shown in online supplementary table 1.

**Table 1:** Description of the characteristics of low-income and middle-income countries included in analyses

Variable	Source	Scale	N	Mean	SD	Minimum	Maximum
CCI	International Center for Equity in Health	0%–100%	80	70.5	13.3	33.5	89.8
Concentration index for CCI	International Center for Equity in Health	−100 to +100	80	5.34	5.10	−1.26	27.84
Slope index for CCI	International Center for Equity in Health	−100 to +100 per cent points	80	19.75	14.72	−6.29	69.75
Control of corruption	World Bank governance indicators	−2.5 to +2.5 z-scores	80	−0.68	0.44	−1.50	0.83
Government effectiveness	World Bank governance indicators	−2.5 to +2.5 z-scores	80	−0.68	0.47	−1.63	0.34
Political stability and absence of violence	World Bank governance indicators	−2.5 to +2.5 z-scores	80	−0.63	0.79	−2.69	0.93
Regulatory quality	World Bank governance indicators	−2.5 to +2.5 z-scores	80	−0.56	0.54	−2.18	0.49
Rule of law	World Bank governance indicators	−2.5 to +2.5 z-scores	80	−0.68	0.46	−1.58	0.42
Voice and accountability	World Bank governance indicators	−2.5 to +2.5 z-scores	80	−0.56	0.66	−2.04	1.04
GDP per capita	World Bank	Per capita GDP (US$, power purchasing parity), log scale	79	8.23	0.89	6.54	9.93
Gini index for income	World Bank	0–100	80	39.92	9.10	16.64	60.97
Country surface area	World Bank	Square kilometres (million)	80	0.49	0.64	0.00	2.97
Country population	World Bank	Inhabitants (million)	80	40.35	132.55	0.16	1144.33

CCI, composite coverage index; GDP, gross domestic product; n, number of countries.

Table 2 shows the unadjusted correlation matrix for all indicators. The six dimensions of governance were positively associated with the CCI, and the association was particularly strong for government effectiveness (see also online supplementary figure 1). GDP per capita was also positively associated with CCI. Better governance—and in particular political stability and absence of violence—was inversely associated with both relative and absolute inequalities. Of the 12 associations tested, only one—between absolute inequality and the voice/accountability indicator—did not achieve significance level. GDP per capita was inversely related to both absolute and relative inequality measures. Country surface area was positively related with inequalities on CCI, while country population was associated with absolute, but not with relative inequalities.

**Table 2:** Matrix correlation between CCI (coverage, CIX and SII), governance indicators, GDP, Gini index, country surface area and population

	CCI	CIX for CCI	SII for CCI	Control of corruption	Government effectiveness	Political stability and absence of violence	Regulatory quality	Rule of law	Voice and accountability	Log GDP per capita	Gini index for income	Country surface area	Country population
CCI	1.00												
CIX for CCI	−0.86	1.00											
	p<0.001												
SII for CCI	−0.80	0.96	1.00										
	p<0.001	p<0.001											
Control of corruption	0.37	−0.31	−0.28	1.00									
	p<0.001	p=0.005	p=0.013										
Government effectiveness	0.57	−0.39	−0.35	0.74	1.00								
	p<0.001	p<0.001	p=0.001	p<0.001									
Political stability and absence of violence	0.52	−0.53	−0.48	0.46	0.46	1.00							
	p<0.001	p<0.001	p<0.001	p<0.001	p<0.001								
Regulatory quality	0.43	−0.30	−0.27	0.69	0.84	0.37	1.00						
	p<0.001	p=0.07	p=0.014	p<0.001	p<0.001	p<0.001							
Rule of law	0.46	−0.37	−0.34	0.85	0.86	0.53	0.80	1.00					
	p<0.001	p<0.001	p<0.001	p<0.001	p<0.001	p<0.001	p<0.001						
Voice and accountability	0.32	−0.26	−0.21	0.59	0.62	0.45	0.66	0.66	1.00				
	p=0.004	p=0.021	p=0.065	p<0.001	p<0.001	p<0.001	p<0.001	p<0.001					
Log GDP per capita	0.63	−0.44	−0.44	0.25	0.54	0.26	0.47	0.38	0.24	1.00			
	p<0.001	p<0.001	p<0.001	p=0.027	p<0.001	p=0.022	p<0.001	p<0.001	p=0.032				
Gini index for income	−0.04	0.04	0.07	0.31	0.10	0.19	0.09	0.13	0.21	−0.11	1.00		
	p=0.712	p=0.717	p=0.563	p=0.005	p=0.401	p=0.093	p=0.447	p=0.246	p=0.057	p=0.344			
Country surface area	−0.20	0.31	0.32	−0.16	−0.04	−0.32	−0.08	−0.08	−0.10	0.01	−0.09	1.00	
	p=0.069	p=0.005	p=0.004	p=0.147	p=0.697	p=0.003	p=0.502	p=0.489	p=0.367	p=0.910	p=0.416		
Country population	−0.09	0.20	0.26	0.02	0.15	−0.17	0.05	0.17	0.13	−0.01	−0.11	0.52	1.00
	p=0.438	p=0.070	p=0.022	p=0.894	p=0.193	p=0.123	p=0.685	p=0.142	p=0.244	p=0.906	p=0.347	p<0.001	

CCI, composite coverage index; CIX, concentration index.; GDP, gross domestic product; SII, slope index of inequality.

Table 3 shows the meta-regression results for the three outcomes. When governance indicators were adjusted for one another, only government effectiveness and political stability/absence of violence remained associated with CCI. When further adjustment was made for country characteristics, only political stability/absence of violence was still associated with CCI, with just a small drop in the magnitude of its coefficient (from 6.7 to 6.3). GDP per capita was strongly and directly associated with the CCI in all models, and surface area became inversely associated with the CCI when controlled for other country characteristics.

**Table 3:** Meta-regression analysis for governance indicators, GDP, Gini index, country population and surface area, and CCI coverage, CIX and SII

		Unadjusted			Adjusted 1*			Adjusted 2†			Adjusted 3‡		
		β (95% CI)	p Value	Adjusted R² (%)	β (95% CI)	p Value	Adjusted R² (%)	β (95% CI)	p Value	Adjusted R² (%)	β (95% CI)	p Value	Adjusted R² (%)
CCI coverage	Control of corruption	**11.2 (4.9 to 17.6)**	**0.001**	12.9	−2.7 (−12.7 to 7.1)	0.596	39.9				–	*–*	52.0
	Government effectiveness	**16.1 (10.8 to 21.4)**	**<0.001**	31.8	**20.1 (9.0 to 31.2)**	**0.001**					–	*–*	
	Political stability and absence of violence	**8.6 (5.4 to 11.9)**	**<0.001**	25.7	**6.7 (3.1 to 10.2)**	**<0.001**					**6.3 (3.6 to 9.1)**	**<0.001**	
	Regulatory quality	**10.8 (5.7 to 15.8)**	***<*0.001**	17.7	−0.1 (−8.9 to 8.7)	0.988					–	*–*	
	Rule of law	**13.1 (7.3 to 18.9)**	**<0.001**	19.9	−6.2 (−19.3 to 7.0)	0.353					–	*–*	
	Voice and accountability	**6.4 (2.0 to 10.7)**	**0.004**	8.9	−2.2 (−7.3 to 2.8)	0.382					–	*–*	
	GDP per capita (log scale)	**9.5 (6.8 to 12.1)**	**<0.001**	39.1				**9.5 (6.9 to 12.2)**	**<0.001**	**41.4**	**8.0 (5.6 to 10.5)**	**<0.001**	
	Gini index for income	−0.1 (−0.4 to 0.3)	0.717	−1.1				0.0 (−0.2 to 0.3)	0.896		–	*–*	
	Country surface area (km^2^, million)	−4.2 (−8.8 to 0.4)	0.071	2.9				**−4.8 (−9.1 to −0.5)**	**0.028**		–	*–*	
	Country population (inhabitants, million)	−0.01 (−0.03 to 0.01)	0.438	−0.5				0.00 (−0.02 to 0.03)	0.668		–	*–*	
Concentration index for CCI	Control of corruption	**−2.2 (−4.2 to −0.2)**	**0.033**	2.8	1.2 (−2.1 to 4.5)	0.457	26.6				–	*–*	51.3
	Government effectiveness	**−3.4 (−5.2 to −1.5)**	**<0.001**	17.0	**−4.2 (−8.1 to −0.3)**	**0.033**					–	*–*	
	Political stability and absence of violence	**−2.5 (−3.6 to −1.4)**	**<0.001**	21.0	**−2.2 (−3.5 to −0.9)**	**0.001**					**−1.4 (−2.4 to −0.4)**	**0.007**	
	Regulatory quality	**−2.1 (−3.7 to −0.4)**	**0.014**	6.5	0.3 (−2.6 to 3.1)	0.861					–	*–*	
	Rule of law	**−2.8 (−4.7 to −1.0)**	**0.004**	9.8	0.6 (−3.9 to 5.0)	0.798					–	*–*	
	Voice and accountability	−1.2 (−2.6 to 0.2)	0.092	−0.4	0.9 (−0.8 to 2.6)	0.288					–	*–*	
	GDP per capita (log scale)	**−2.3 (−3.1 to −1.4)**	**<0.001**	35.8				**−2.3 (−3.1 to −1.5)**	*<*0.001	47.7	**−2.0 (−2.8 to −1.2)**	**<0.001**	
	Gini index for income	0.0 (−0.1 to 0.1)	0.537	−0.5				0.0 (−0.1 to 0.1)	0.553		–	*–*	
	Country surface area (km^2^, million)	**1.9 (0.5 to 3.3)**	**0.009**	8.4				**1.9 (0.5 to 3.2)**	**0.007**		**1.6 (0.5 to 2.7)**	**0.007**	
	Country population (inhabitants, million)	0.01 (−0.01 to 0.02)	0.076	5.1				0.00 (−0.01 to 0.01)	0.621		–	*–*	
Slope index for CCI	Control of corruption	**−8.8 (−16.1 to −1.6)**	**0.017**	5.8	1.6 (−10.9 to 14.1)	0.799	21.4				–	*–*	39.0
	Government effectiveness	**−11.0 (−17.8 to −4.2)**	**0.002**	10.8	−9.1 (−23.5 to 5.4)	0.212					–	*–*	
	Political stability and absence of violence	**−8.8 (−12.5 to −5.1)**	**<0.001**	22.7	**−8.2 (−12.7 to −3.7)**	**0.001**					**−5.3 (−8.9 to −1.7)**	**0.005**	
	Regulatory quality	**−7.1 (−13.2 to −1.1)**	**0.021**	5.0	−0.1 (−11.2 to 10.9)	0.983					–	*–*	
	Rule of law	**−10.3 (−17.1 to −3.5)**	**0.003**	9.2	0.6 (−16.0 to 17.2)	0.943					–	*–*	
	Voice and accountability	−4.3 (−9.3 to 0.8)	0.095	1.9	3.3 (−3.1 to 9.7)	0.308					–	*–*	
	GDP per capita (log scale)	**−7.2 (−10.8 to −3.7)**	**<0.001**	16.4				**−7.2 (−10.6 to −3.9)**	**<0.001**	26.4	**−6.3 (−9.3 to −3.2)**	**<0.001**	
	Gini index for income	0.1 (−0.3 to 0.5)	0.576	−0.9				0.1 (−0.2 to 0.4)	0.585		–	*–*	
	Country surface area (km^2^, million)	**7.2 (2.3 to 12.1)**	**0.004**	9.2				**6.3 (1.0 to 11.5)**	**0.020**		**5.2 (0.9 to 9.4)**	**0.018**	
	Country population (inhabitants, million)	**0.03 (0.00 to 0.05)**	**0.020**	**6.0**				0.01 (−0.01 to 0.04)	0.311		**–**	***–***	

Bold numbers indicate statistically significant associations.

*Adjusted for other governance indicators.

†Adjusted for non-governance indicators (GDP, Gini, surface area and population).

‡Adjusted for all variables with p<0.05 in the adjusted models 1 and 2.

β, meta-regression coefficient; CCI, composite coverage index; CIX, concentration index; GDP, gross domestic product; R², coefficient of determination; SII, slope index of inequality.

Regarding relative inequality (table 3), there were inverse associations between the CIX and all the six governance indicators in the unadjusted models. Political stability/absence of violence was the only governance indicator to remain significant in the adjusted models. Regarding country characteristics, inequalities were smaller in countries with higher GDP per capita, but increased with population size. Countries with large surface areas had slightly higher inequalities, but only in the model adjusted for other country characteristics.

The governance indicator with strongest inverse associations with absolute inequality (table 3) was political stability/absence of violence, with p values of 0.001 when adjusted for other governance indicators and 0.005 when also adjusted for country characteristics. No other governance indicators had significant associations in the adjusted models. Direct associations with absolute inequalities were found for population size and surface area, while the association with GDP was inverse.

The coefficient of determination (R²) presented in table 3 showed, in the final adjusted model, that 52% of the variability of the CCI at the country level is explained by political stability/absence of violence and GDP per capita. Regarding inequality measures, political stability/absence of violence, GDP per capita and country surface area explained 51% of relative inequality variability (CIX) and 39% of absolute inequality variability (SII).

**Figure 1:**
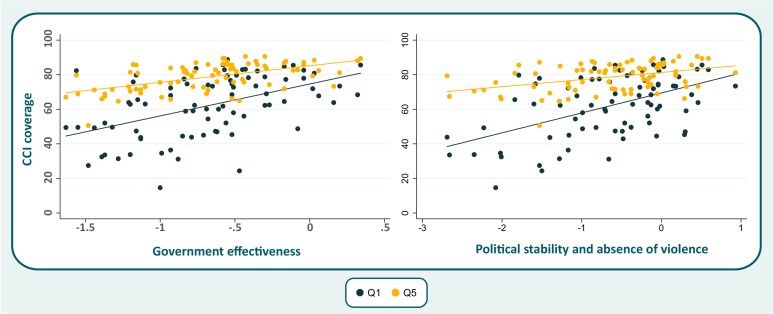
Scatter plot for selected governance indicators with composite coverage index (CCI), by wealth quintiles (Q1—poorest and Q5—richest).

**Figure 2:**
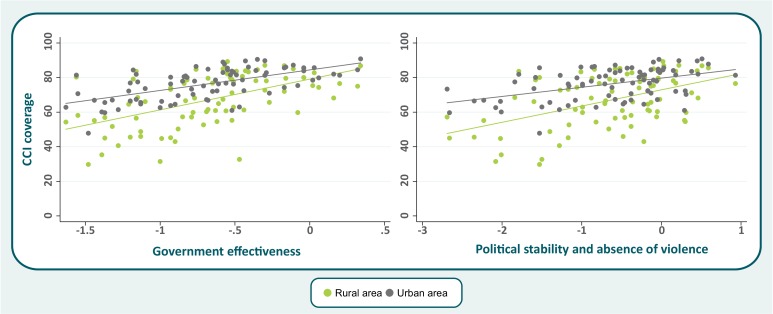
Scatter plot for selected governance indicators with composite coverage index (CCI), by area of residence.

Government effectiveness and political stability/absence of violence were the two best predictors of coverage outcomes among the six governance indicators. In figure 1, we show that these two indicators are more strongly associated with coverage in the poorest (Q1) than in the richest (Q5) quintile. Stronger associations were also found for rural than for urban women and children (figure 2).

## Discussion

Our unadjusted ecological analyses show that good governance is consistently associated with both higher national levels and lower magnitudes of within-country inequalities in RMNCH coverage. When the six governance indicators are adjusted for each other and for national characteristics, only political stability and absence of violence remained associated with higher national coverage and lower inequalities. Our results support the recent literature showing the negative impact of conflict and humanitarian emergencies on several RMNCH outcomes.^23 24^ The reasons behind this association vary, but they are closely related to the disruption of preventive and curative services, as well as to specific crises such as lack of food or shelter.^25 26^ In the conflict literature, we were unable to find analyses of how such crises affect inequalities. Our analyses showed that inequalities were directly associated to conflict, and that political stability and absence of violence, as well as government effectiveness, were more strongly associated with coverage among the poorest than among the richest. This finding suggests that the wealthy may benefit from safety nets that make them less dependent from the state-provided services, thus allowing rich families to offset poor governance.

Our results on an association between governance and coverage are apparently contradictory to those reported by Alkenbrack *et al*,^15^ who relied on the same data sources for 74 LMICs. Instead of a CCI, they investigated four outcomes: contraceptive prevalence, DFPS, antenatal care and delivery in a health facility. The six dimensions of governance were summarised through PCA, resulting in a single factor that was heavily driven by the rule of law and government effectiveness dimensions. Had our analyses relied on a similar summary index, they would be unlikely to find an association, because neither rule of law nor government efficiency was correlated with coverage in the adjusted models. By keeping the six dimensions of governance separate, we were able to show an association between conflict and coverage. Our results are in agreement with the analyses by Arsenault *et al* 16 showing that the political stability and absence of violence indicator was directly associated with coverage and inversely related to inequalities for vaccine coverage in countries supported by the Gavi Alliance.

Among factors other than governance indicators, our analyses found that GDP per capita was directly associated with coverage and inversely related to inequalities, whereas geographical surface area showed opposite trends. Although the association of GDP with RMNCH indicators has been repeatedly confirmed by the literature,^7–9^ fewer studies report on the association with surface area. A modelling study found that country population size found no impact on national health-related performance.^27^ Arsenault *et al* 16, similar to our findings, reported an association between surface area and immunisation coverage and inequalities in specific countries.

Our analyses found that, once controlled by GDP and surface area, country population was not significantly associated with coverage or inequality, suggesting that geographical distance rather than number of inhabitants may be an important challenge to high and equitable coverage. The most obvious explanation is that large countries may face difficulties on delivering services to remote areas, thus leading to lower coverage and greater inequality.^16^

Ecological, cross-sectional analyses such as ours cannot establish the directionality of an association. An extensive literature on investing in health proposes that the health–wealth interaction works in both directions,^28 29^ and that good health may improve economic productivity and therefore GDP and related indicators. In our analyses, it would seem more likely that contextual variables would affect coverage with interventions delivered to women and children, rather than the opposite. Nevertheless, high intervention coverage could be a marker for health systems that are effective in improving reproductive and child health, and the health of the whole population, in which case a bidirectional effect could be postulated.

Governance matters for the achievement of universal health worldwide. Despite the countries being sovereign states, with their own rules and policies, our results showed that good governance, especially avoiding unnecessary conflicts and promoting political stability, might be important towards coverage of basic health interventions for RMNCH. Mechanisms in which better governance influences global health should be further investigated in country-case studies, for example. Also, governance at the global level should be strengthened.^30^ A better governance at the global level demands some efforts from a variety of global actors. These actors include country governments, donors and global institutions such as WHO and Unicef, among others.^30^ Governance should be reinforced as an issue of supranational interest, towards better responses to complex and serious global health problems.^30^

The strengths of our analyses include the use of DHS and MICS data sets, both of which are designed to be nationally representative, have consistent sampling schemes and comparable questionnaires. Reanalyses of the microdata from these surveys at our International Center for Equity in Health ensure that the estimates are reliable and comparable, through standardised data extraction and recoding. Reliance on a composite indicator for coverage along the RMNCH continuum of care results in less random variability and greater precision than would result from analyses of standalone coverage indicators. The CCI is remarkably robust in terms of which coverage variables are included and of which weights are used for each indicator.^6^ Previous analyses have shown that the inclusion of additional coverage indicators did not make any significant difference to the CCI either in terms of how well it summarises health intervention coverage or predicts child health outcomes such as malnutrition and mortality.^6^

Our analyses also have limitations. The governance indicators proposed by the World Bank have been criticised because of their lack of a valid underlying construct,^31 32^ their focus on the private sector (especially for regulatory quality, defined in terms of how governments promote the private sector) and by lack of details on how a large amount of information was compiled and combined into the six dimensions of governance. It is reassuring that our adjusted analyses found no evidence of an association between coverage and the controversial indicator on regulatory quality. Another limitation is that countries undergoing conflict and humanitarian emergencies may be less likely to have had national surveys due to security reasons; nevertheless, from the list of 33 fragile states according to the World Bank,^33^ we included 20, or about two-thirds, in the present analyses (data not shown). The inclusion of countries from LMICs was based on data availability from standardised surveys, which are not carried out in high-income countries. Our analyses included around 60% of the 139 countries listed as low-income and middle-income: 84% of low-income countries, 67% of lower-middle-income countries and only 33% of the upper-middle-income countries (data not shown). The latter—including Brazil, China and Mexico, for example—are less likely to participate in the international survey collaborations that provided data for our analyses.

Our results on RMNCH coverage are largely consistent with studies that relate governance to lower mortality^11 12^ and better nutrition^14^ among young children, and suggest that ensuring higher coverage is a potential link between governance and favourable health outcomes. Unlike previous analyses, we were able to provide evidence that good governance is more likely to improve coverage among the poor than among the rich, and thus contribute to national-level progress towards leaving no one behind.

## Conclusion

Our findings show that political stability and absence of violence, as well as per capita GDP, are directly associated with RMNCH coverage and inversely related to inequalities, and that country surface area is directly associated with inequalities. Our results reinforce the importance of good governance, particularly absence of violence and conflict, as drivers of the health of mothers and children. In a world where 33 countries are currently reported to be in conflict situations, global efforts to increase good governance practices are needed to reduce mortality and improve general health for the entire population.
