# Supplementary material for: Is governance, gross domestic product, inequality, population size or country surface area associated with coverage and equity of health interventions? Ecological analyses of cross-sectional surveys from 80 countries
Source: BMJ Glob Health. 2017 Oct 31;2(4):e000437. doi: 10.1136/bmjgh-2017-000437 (PMC5717925; doi:10.1136/bmjgh-2017-000437)

Supplementary Box 1 – Definition of governance indicators

| Indicator | Definition |
|---|---|
| Voice and accountability | Capturing perceptions of the extent to which a country's citizens are able to participate in selecting their government, as well as freedom of expression, freedom of association, and a free media |
| Political stability and absence of violence | Capturing perceptions of the likelihood that the government will be destabilized or overthrown by unconstitutional or violent means, including politically-motivated violence and terrorism |
| Government effectiveness | Capturing perceptions of the quality of public services, the quality of the civil service and the degree of its independence from political pressures, the quality of policy formulation and implementation, and the credibility of the government's commitment to such policies |
| Regulatory quality | Capturing perceptions of the ability of the government to formulate and implement sound policies and regulations that permit and promote private sector development |
| Rule of law | Capturing perceptions of the extent to which agents have confidence in and abide by the rules of society, and in particular the quality of contract enforcement, property rights, the police, and the courts, as well as the likelihood of crime and violence |
| Control of corruption | Capturing perceptions of the extent to which public power is exercised for private gain, including both petty and grand forms of corruption, as well as "capture" of the state by elites and private interests |

Source: Kaufmann D, Kraay A, Mastruzzi M. The worldwide governance indicators: methodology and analytical issues. Hague Journal on the Rule of Law. 2011;3(2):220-46.

Supplementary Table 1 – Surveys included and estimate, concentration index and slope index of inequalities for composite coverage index.

| Country | Year | Source | CCI (%) | CIX for CCI | SII for CCI (pp) |
|---|---|---|---|---|---|
| Albania | 2008 | DHS | 86.8 | 0.55 | 2.97 |
| Armenia | 2010 | DHS | 85.7 | 1.04 | 5.49 |
| Azerbaijan | 2006 | DHS | 64.3 | 4.29 | 17.18 |
| Bangladesh | 2014 | DHS | 70.6 | 4.87 | 21.50 |
| Belize | 2011 | MICS | 80.1 | 2.68 | 13.40 |
| Benin | 2011 | DHS | 57.6 | 6.88 | 24.50 |
| Bolivia | 2008 | DHS | 71.3 | 5.59 | 25.06 |
| Bosnia and Herzegovina | 2011 | MICS | 85.2 | 0.97 | 5.18 |
| Burkina Faso | 2010 | DHS | 64.6 | 7.96 | 31.25 |
| Burundi | 2010 | DHS | 66.8 | 2.90 | 12.10 |
| Cambodia | 2014 | DHS | 80.4 | 2.87 | 14.47 |
| Cameroon | 2011 | DHS | 59.2 | 14.04 | 49.90 |
| CAR | 2010 | MICS | 44.8 | 15.26 | 42.18 |
| Chad | 2014 | DHS | 33.5 | 11.19 | 23.58 |
| Colombia | 2010 | DHS | 84.1 | 2.01 | 10.70 |
| Comoros | 2012 | DHS | 62.1 | 6.64 | 25.75 |
| Congo Brazzaville | 2011 | DHS | 72.8 | 5.02 | 22.90 |
| Congo Democratic Republic | 2013 | DHS | 59.2 | 6.95 | 25.56 |
| Costa Rica | 2011 | MICS | 89.8 | 0.65 | 3.63 |
| Cote dIvoire | 2011 | DHS | 55.5 | 9.03 | 31.16 |
| Dominican Republic | 2013 | DHS | 82.1 | 0.15 | 0.78 |
| Egypt | 2014 | DHS | 79.1 | 1.60 | 7.90 |
| Ethiopia | 2011 | DHS | 37.4 | 18.70 | 43.64 |
| Gabon | 2012 | DHS | 68.8 | 4.32 | 18.50 |
| Gambia | 2013 | DHS | 63.9 | 3.00 | 11.92 |
| Ghana | 2014 | DHS | 67.9 | 3.68 | 15.63 |
| Guinea | 2012 | DHS | 46.1 | 12.92 | 37.37 |
| Guinea Bissau | 2006 | MICS | 52.1 | 10.50 | 34.00 |
| Guyana | 2009 | DHS | 72.6 | 1.57 | 7.13 |
| Haiti | 2012 | DHS | 57.5 | 6.43 | 23.16 |
| Honduras | 2011 | DHS | 83.7 | 1.76 | 9.23 |
| India | 2005 | DHS | 63.9 | 10.33 | 41.32 |
| Indonesia | 2012 | DHS | 80.4 | 3.33 | 16.78 |
| Iraq | 2011 | MICS | 74.6 | 3.88 | 18.16 |
| Jordan | 2012 | DHS | 84.1 | 0.62 | 3.29 |
| Kazakhstan | 2010 | MICS | 86.8 | 1.43 | 7.76 |
| Kenya | 2014 | DHS | 75.8 | 5.71 | 26.95 |
| Kosovo | 2013 | MICS | 84.1 | 2.10 | 11.25 |
| Kyrgyzstan | 2014 | MICS | 77.4 | 1.62 | 7.92 |
| Lao | 2011 | MICS | 61.4 | 12.43 | 48.46 |
| Lesotho | 2014 | DHS | 79.2 | 2.85 | 14.10 |

| | | | | | |
|---|---|---|---|---|---|
| Liberia | 2013 | DHS | 61.4 | 5.30 | 20.42 |
| Macedonia | 2011 | MICS | 86.0 | 0.50 | 2.66 |
| Madagascar | 2008 | DHS | 63.2 | 11.20 | 43.74 |
| Malawi | 2013 | MICS | 80.1 | 1.46 | 7.34 |
| Maldives | 2009 | DHS | 79.9 | -1.26 | -6.29 |
| Mali | 2012 | DHS | 49.4 | 13.16 | 39.46 |
| Mauritania | 2011 | MICS | 52.9 | 11.78 | 38.11 |
| Moldova | 2012 | MICS | 87.5 | -0.63 | -3.46 |
| Mongolia | 2010 | MICS | 87.3 | 0.98 | 5.33 |
| Montenegro | 2013 | MICS | 77.5 | 1.87 | 8.98 |
| Mozambique | 2011 | DHS | 60.2 | 9.88 | 36.31 |
| Namibia | 2013 | DHS | 79.1 | 1.68 | 8.30 |
| Nepal | 2014 | MICS | 68.5 | 6.39 | 27.49 |
| Niger | 2012 | DHS | 56.3 | 8.92 | 30.69 |
| Nigeria | 2013 | DHS | 43.3 | 27.84 | 69.75 |
| Pakistan | 2012 | DHS | 62.2 | 11.11 | 42.50 |
| Panama | 2013 | MICS | 80.5 | 4.23 | 21.87 |
| Peru | 2012 | DHS | 83.9 | 2.47 | 13.13 |
| Philippines | 2013 | DHS | 76.9 | 3.60 | 17.50 |
| Rwanda | 2014 | DHS | 75.9 | 1.88 | 8.93 |
| Sao Tome and Principe | 2008 | DHS | 74.7 | 1.83 | 8.56 |
| Senegal | 2015 | DHS | 65.7 | 5.65 | 23.30 |
| Sierra Leone | 2013 | DHS | 66.8 | 2.85 | 11.90 |
| Sudan | 2010 | MICS | 49.6 | 13.89 | 42.55 |
| Swaziland | 2010 | MICS | 80.1 | 2.53 | 12.63 |
| Syria | 2006 | MICS | 77.3 | 3.80 | 18.37 |
| Tajikistan | 2012 | DHS | 74.0 | 0.99 | 4.61 |
| Tanzania | 2010 | DHS | 71.1 | 6.62 | 29.33 |
| Timor Leste | 2009 | DHS | 59.2 | 7.93 | 28.87 |
| Togo | 2013 | DHS | 57.2 | 7.71 | 27.39 |
| Tunisia | 2011 | MICS | 84.7 | 1.54 | 8.18 |
| Turkmenistan | 2006 | MICS | 80.6 | -0.96 | -4.83 |
| Uganda | 2011 | DHS | 65.0 | 5.04 | 20.31 |
| Ukraine | 2012 | MICS | 88.0 | 0.75 | 4.15 |
| Uzbekistan | 2006 | MICS | 83.2 | 1.13 | 5.90 |
| Vietnam | 2013 | MICS | 87.8 | 2.19 | 12.25 |
| Yemen | 2013 | DHS | 51.6 | 13.98 | 44.05 |
| Zambia | 2013 | DHS | 75.6 | 4.85 | 23.06 |
| Zimbabwe | 2014 | MICS | 80.6 | 2.52 | 12.71 |

Supplementary Figure 1 – Scatter plots for composite coverage index (coverage and inequality measures [concentration index and slope index of inequality]) and governance indicators.

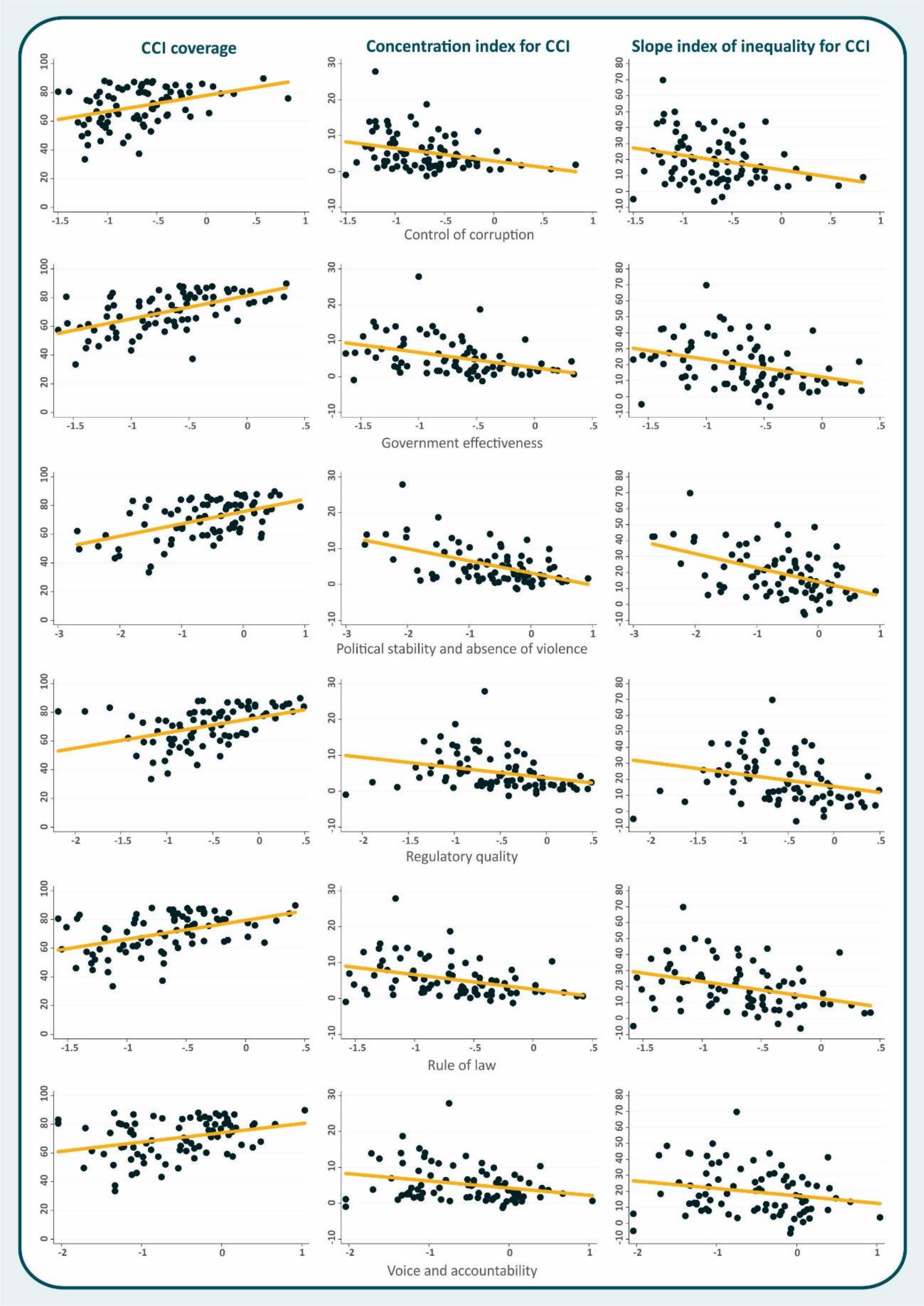

Supplement: Supplementary file 1 [file bmjgh-2017-000437supp001.pdf]
